# Deep Learning Methods for Improving Pollen Monitoring

**DOI:** 10.3390/s21103526

**Published:** 2021-05-19

**Authors:** Elżbieta Kubera, Agnieszka Kubik-Komar, Krystyna Piotrowska-Weryszko, Magdalena Skrzypiec

**Affiliations:** 1 Department of Applied Mathematics and Computer Science, University of Life Sciences in Lublin, ul. Głęboka 28, 20-950 Lublin, Poland; 2 Department of Botany and Plant Physiology, University of Life Sciences in Lublin, Akademicka 15, 20-950 Lublin, Poland; krystyna.piotrowska@up.lublin.pl; 3 Institute of Mathematics, Maria Curie-Sklodowska University, pl. Marii Curie-Skłodowskiej 1, 20-031 Lublin, Poland; mskrzypiec@hektor.umcs.lublin.pl

**Keywords:** pollen monitoring, classification, deep neural networks

## Abstract

The risk of pollen-induced allergies can be determined and predicted based on data derived from pollen monitoring. Hirst-type samplers are sensors that allow airborne pollen grains to be detected and their number to be determined. Airborne pollen grains are deposited on adhesive-coated tape, and slides are then prepared, which require further analysis by specialized personnel. Deep learning can be used to recognize pollen taxa based on microscopic images. This paper presents a method for recognizing a taxon based on microscopic images of pollen grains, allowing the pollen monitoring process to be automated. In this research, a deep CNN (convolutional neural network) model was built from scratch. Publicly available deep neural network models, pre-trained on image data (not including microscopic pictures), were also used. The results show that even a simple deep learning model produces quite good results when the classification of pollen grain taxa is performed directly from the images. The best deep learning model achieved 97.88% accuracy in the difficult task of recognizing three types of pollen grains (birch, alder, and hazel) with similar structures. The derived models can be used to build a system to support pollen monitoring experts in their work.

## 1. Introduction

Pollen grains are among the main sources of allergens [1]. Information on the amounts of these allergens in the air comes from pollen monitoring, where the volumetric method using Hirst-type samplers is employed [2]. This method is recommended by the International Association for Aerobiology, and is commonly used in pollen monitoring centers across Poland. Hirst-type samplers actively suck in pollen grains from the air and trap them on sticky tape. Each piece of tape must be analyzed by appropriately trained staff, who identify and count the pollen grains under a microscope. This is a difficult and time-consuming task, because the differences in the morphological structures of pollen grains of some taxa are very small. Detecting the characteristic features of a particular taxon can be additionally hindered due to the specific location of a grain on the tape. Therefore, there is a need to automate, or at least facilitate, this process.

During the Polish spring, birch (*Betula*) pollen shows the strongest allergenic properties [3,4]; it achieves the highest total concentration over the pollen season and the highest daily amount of pollen in the air [4]. Hazel (*Corylus*) and alder (*Alnus*) belong to the same family (Betulaceae), and cause allergic cross-reactions [3,5]. The pollen seasons of these plants partially overlap, and thus their pollen grains may be recorded during the same time. This research aims to distinguish between these three investigated types of pollen (birch, alder, and hazel) based on their microscope images.

There are many different methods for collecting and recognizing taxa in pollen monitoring. These include, among others: automated multispectral imaging flow cytometry used in combination with deep learning [6], the metabarcoding method of species differentiation based on DNA data [7], and labeling procedures based on the combination of fluorescein (FDA) and propidium iodide (PI), together with automatic counting of pollen grains [8]. However, in Poland, the above-mentioned taxon evaluation—using slides obtained via the volumetric method, and their analysis under an optical microscope—is a typical way of compiling monitoring data.

In this paper, deep learning (deep neural networks) is used to recognize pollen taxa based on microscopic images. The deep learning methods make it possible to classify objects directly from pictures, so no feature extraction or additional annotation is needed before building the model. Thus, the pollen expert’s effort during the data preparation can be minimized and limited to microscopic image acquisition.

The precision of the recognition and classification depends on many factors. The structural differences between the objects seem to be the main ones. In our study, the high similarity between birch, hazel, and alder pollen grains complicates the problem.

### 1.1. Literature Review

Many previous studies on taxon discrimination were carried out via image-based classification using neural networks. Li and Flenley [9] considered the pollen grains of four plant types indigenous to New Zealand. The authors themselves admitted that these grains could be easily differentiated by their shapes, but in the article they distinguished them only by their texture features, obtaining 100% accuracy. France et al. [10] were the first to create an automated system to detect and classify *Polemonium caeruleum*, *Nymphaea alba*, and *Crataegus monogyna* pollen grains. They obtained an average classification success rate of 83%. The plants mentioned above produce pollen grains with different structures, particularly their aperture structures and exines. Tello-Mijares et al. [11] addressed the problem of automated analysis of pollen material collected in Mexico (12 plant taxa typical for this area), obtaining around a 96% rate of pollen recognition. Most of these pollen grains were fairly easy to recognize, but there were also grains similar to one another.

Deep learning methods have been applied to the recognition of pollen grains (see, among others, [12,13,14,15]). The AlexNet and SmallerVGGNet models were used in [12] for the Pollen13K database, resulting in average F-scores of 0.87 and 0.85, respectively.

In [13], the authors used a publicly available dataset of 23 types of Brazilian Savannah pollen [16]. They obtained impressive results: a classification rate of 97.2%, with a low standard deviation of 0.9%, for one of the three implemented models. In their more recent article [14], convolutional neural networks were used to classify 46 pollen types. The authors used the pre-trained AlexNet network as a feature extractor, and then applied a linear discriminant classifier, obtaining 97% accuracy. A pre-trained CNN network has been frequently used as a feature extractor in other studies. A comparison can be found in [17].

Fine-tuning of the pre-trained CNNs (8 different architectures) was applied in [15] to the pollen grains of 73 savannah plants. They obtained the best results (almost 96% accuracy) using the DenseNet model in the 5-fold cross-validation procedure.

### 1.2. The Aim of the Study

This research aims to indicate the classifier building method that produces the best results in identifying birch, alder, and hazel pollen grains. An additional benefit of this study is a set of microscopic images of pollen grains of these three taxa obtained from reference slides. It is worth mentioning that none of the publicly available datasets of microscope images of pollen grains, presented in [18], contain images of birch pollen grains.

The newly created database was used to build and validate the performance of the following classifiers: (1) the CNN trained from scratch—SimpleModel—and (2) the modification of previously trained deep learning models—AlexNet [19], ResNet [20], VGG [21], DenseNet [22], SqueezeNet [23], and InceptionV3 [24]. The broad set of CNN architectures made it possible to test the effectiveness of different deep networks in recognizing pollen grains directly from microscopic images. To our best knowledge, no research other than that presented in this paper has used SqueezeNet for this topic. It is worth mentioning that, according to our experimental results, this model is faster than ResNet, VGG, DenseNet, and InceptionV3. This property of SqueezeNet was also observed in [25], where the author measured the amount of pollen introduced into the hive in the pollen bags of bees.

The presented research assessed whether the use of the additional database containing the microscope images of the pollen grains during the models’ pre-training could increase the accuracy of taxon recognition for the ABCPollen database. The Pollen13K database [12] is currently the largest publicly available set of microscopic images of pollen grains [18], and is used in this research for the pre-training of some models. The results obtained for these models have been compared with the results of the classifiers pre-trained with the ImageNet database [26], which does not contain microscopic images. This comparison presumably has not been reported before in any other research article on using deep networks to recognize pollen grains.

The results presented in this paper prove that it is justified to use deep learning methods in pollen recognition.

## 2. Materials and Methods

### 2.1. Dataset

This study was conducted based on reference slides of pollen grains of the *Alnus glutinosa*, *Betula pendula*, and *Corylus avellana* taxa. In the following part of this paper, only the generic names of the studied species are used. The pollen grains analyzed in this study differ primarily in their shape and number of pores: the alder pollen grain has five pores—sometimes four or six—whereas the birch and hazel pollen grains have only three (only two are occasionally visible, depending on their position). Unlike the birch pollen grain, the hazel pollen grain is more triangular in a polar view, and has less protruding pores. The studied pollen grains also differ in oncus size. Microscopic visibility of pores and onci greatly depends on the position of the grain on the tape. During manual observation, the researcher can control the microscope’s focus setting in order to observe the grain at different depths, and thus better perceive the characteristic structural elements. When analyzing a single microscope image, it is much more difficult to describe these characteristics correctly.

**Figure 1:**
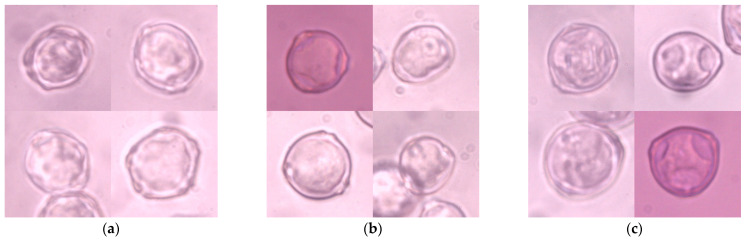
Examples of microscope images of pollen grains of the studied taxa: (**a**) *Alnus*; (**b**) *Betula*; (**c**) *Corylus*.

Four hundred and forty-one 1024 × 786 px microscopic images were captured using a Nikon Eclipse E400 (Nikon Corporation, Tokyo, Japan) biological microscope at a magnification of 600× and labeled manually. These images were cropped manually to 200 × 200 px, which relates to the original size of a single pollen grain in the picture, and constitutes a collection of 1274 images (Figure 1). Each item represented one pollen grain in the center of the image, which might partially overlap with other grains of the same taxon and might be out of focus. There were no multi-label objects in this collection, which made the labeling procedure much more straightforward. There were 406 images of *Alnus* grains, 435 images of *Betula* grains, and 433 images of *Corylus* grains. This dataset was called ABCPollen—where A stands for *Alnus*, B for *Betula*, and C for *Corylus*—and was used as an input for deep learning models.

### 2.2. The CNN Trained from Scratch—Simple Model

**Figure 2:**
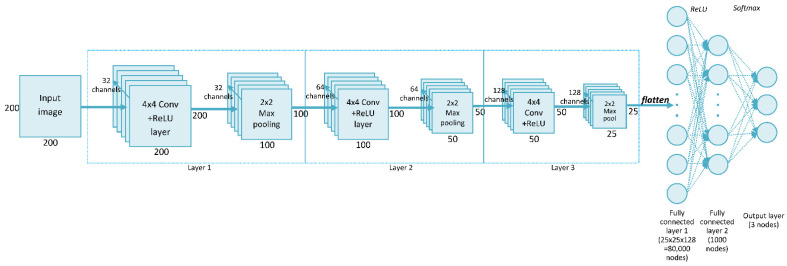
SimpleModel architecture.

The convolutional neural network (CNN) is a widely used deep neural network architecture in multi-object recognition from images [27]. This network can perform classification directly from the picture, so no feature extraction is needed before building the model. Our network, called SimpleModel, trained on data from the ABCPollen dataset, was created during the initial stage of this study. The proposed method was a CNN consisting of three convolutional layers, each with 4 × 4 filters. Figure 2 shows the structure of SimpleModel, which is a modification of the model presented in the CNN tutorial [28]. In relation to the original structure given in this tutorial, the following changes were made in this study: the input image dimensions were set to 200 × 200 px, three convolutional layers were used, and a 4 × 4 filter was selected. This filter size was chosen experimentally, as it gave the highest accuracy compared to results for 3 × 3 and 5 × 5 filters (Table S2, Supplementary Materials). An Adam optimizer was used to train the network [29].

The SimpleModel training process was performed using 1224 randomly chosen images, while the test set contained the remaining 50 samples of ABCPollen data. The number of test items was determined by the small number of images in the ABCPollen database. The model built from scratch needed as many samples for training as possible.

### 2.3. The Modification of Previously Trained Deep Learning Models—Transfer Learning

Deep neural network models require a large amount of training data and long training in order to achieve satisfactory classification quality [30]. Therefore, so-called transfer learning—which involves networks already trained on some large dataset—is frequently used in practice. The idea of using the weights of neural networks trained on other data was described in [31], while the mathematical basis and the geometrical model for the transfer learning of neural networks had been given even 15 years earlier [32]. There are two types of transfer learning:

1. Feature extraction—uses a pre-trained CNN network as a feature extractor by removing the last fully connected layer. This layer performs the role of a classifier. Creating it anew and then training the network to determine only the weights of this new layer is equivalent to building a classifier for the new classes.
2. Fine-tuning—also uses a pre-trained network. Similarly to feature extraction, the last fully connected layer is removed and replaced by a new layer with as many output neurons as classes in the target dataset. Then the entire network is trained (fine-tuned). For all layers, except the last one, the training starts with weights determined during the pre-training.

Theoretically, if the set of images used to train the network is not too large, the former approach (feature extraction) is better. On the other hand, when the objects used for network pre-training have a different character than the objects we want to use to build the target model (e.g., photographs, microscopic images), better classification results will be achieved by using the latter approach (fine-tuning) [32].

By applying the above modifications, we obtained models that distinguished the studied taxa. For this purpose, freely available network architectures such as AlexNet, ResNet, VGG, SqueezeNet, DenseNet, and InceptionV3 were applied. We propose the following names for obtained classifiers, where the label “Model” stands for the used CNN:

- Type 1—Orig_Model—the original model, pre-trained on image data from the ImageNet dataset [26]: Orig_AlexNet, Orig_ResNet, Orig_VGG, Orig_SqueezeNet, Orig_DenseNet, and Orig_InceptionV3;
- Type 2—ScratchPollen13K_Model—a model trained on microscope images from the Pollen13K dataset [12], providing greater similarity of the data from the pre-training and fine-tuning of the network. The models ScratchPollen13K_AlexNet and ScratchPollen13K_ResNet were derived in this fashion;
- Type 3—FinetunedPollen13K_Model—a model pre-trained on pictures from the ImageNet dataset and fine-tuned using Pollen13K data: FinetunedPollen13K_AlexNet and FinetunedPollen13K_ResNet.

Each of the models was subsequently fine-tuned using ABCPollen data. Additionally, the AlexNet- and ResNet-based models were built by removing the last layer from the pre-trained network and using the remaining part of the network as a feature extractor.

The Pollen13K dataset [12] consists of more than 12,000 sample microscopic images of pollen grains, and about 1000 such images of pollution particles. This dataset contains photos of *Corylus avellana* pollen grains (well-developed and anomalous), well-developed *Alnus* pollen grains, Cupressaceae pollen grains, and debris.

The test set composed of 1/10 of the entire ABCPollen dataset, and was randomly selected for each model separately. The remaining objects from this dataset formed the training set. In this case, the training set might include fewer items than the set used for the training of SimpleModel. This seems to be enough for the pre-trained CNNs; thus, the training time can be reduced, which is an important factor in learning for this kind of model.

The classification quality of the selected models was additionally verified by applying a three times tenfold cross-validation (3 × 10CV) design. The long time required to train the models resulted in a limited number of 10CV repetitions. For the 3 × 10CV procedure, the Type 1 models were selected (Orig_), because no significantly better classification accuracy was achieved during fine-tuning of the models using Pollen13K data (the Type 2 and 3 models).

The classification accuracy of the models trained according to the 10CV procedure was verified based on the average accuracy from epochs 91–100 (the last ten epochs of the learning process) and all 3 repetitions. This averaging was chosen in order to better assess the classifiers’ performance when the accuracy was still changing in the final epochs of the training process. Moreover, the accuracy variation expressed as the mean of the three standard deviation values (for each of the repetitions) was presented. The individual accuracy values were determined for the entire dataset as the sum of the number of correctly identified objects in the particular folds divided by the total number of cases.

In this part of the study, no network models were built from scratch for ABCPollen data, due to the low number of objects in this dataset (1274 items). Furthermore, the network trained from scratch produced less satisfactory results for the Pollen13K dataset, relative to those obtained from the network pre-trained on ImageNet data and fine-tuned using Pollen13K (Table S1; Supplementary Materials).

## 3. Results

**Figure 3:**
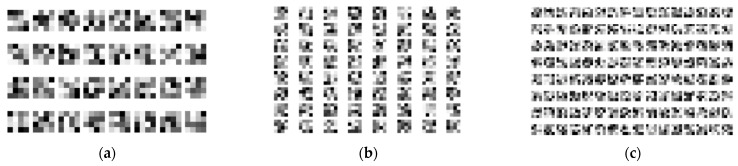
The sets of convolutional filters of size 4 × 4 after 100 epochs of training SimpleModel: (**a**) for the first layer; (**b**) for the second layer; (**c**) for the third layer.

**Figure 4:**
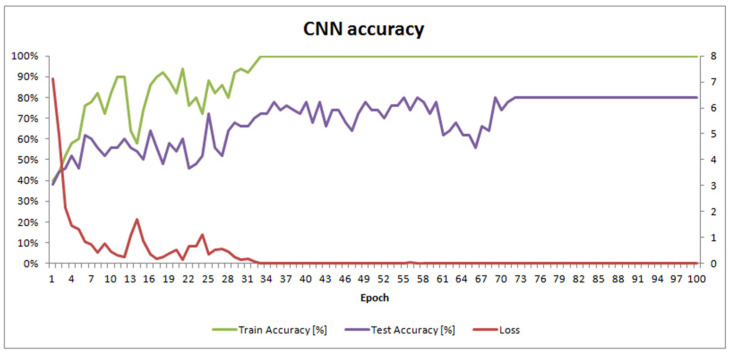
SimpleModel accuracy and loss over the training time.

One hundred epochs of training were performed to train SimpleModel. This took 3 h and 18 min on a mobile workstation equipped with 32 GB of memory and an Intel^®^ Core™ i7 (Intel, Santa Clara, CA, USA) processor with 4 cores, 8 threads, and a 2.4 GHz clock (HP EliteBook 8570w). The final masks of convolutional filters in each layer are shown in Figure 3. SimpleModel’s accuracy changes over time are presented in Figure 4.

SimpleModel’s accuracy results (Table S2; Supplementary Materials) were satisfactory, with an accuracy of 80%. However, we wondered if we would have obtained better results using pre-trained models. Freely available models from the PyTorch library [33] were built using the ImageNet dataset, which contains objects other than microscopic images. We also used the Pollen13K dataset for pre-training, expecting this to improve the results.

The pre-trained AlexNet and ResNet networks were used consecutively in two forms: as feature extractors, and as the starting point for fine-tuning. The CNNs, where the pre-trained network acts as a feature extractor, were compared with the fine-tuned models. The results show that the fine-tuning of the entire network achieves models better fitted to the task at hand. Given the above, the other network architectures were not used as feature extractors, but rather for fine-tuning. The results obtained for the investigated models are contained in Table 1.

**Figure 5:**
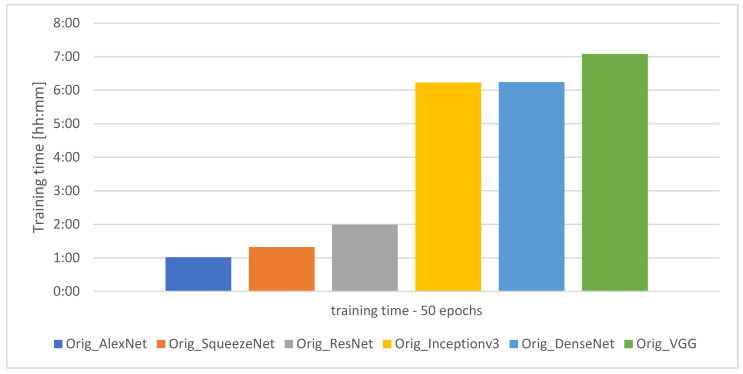
Training time for the individual models.

The graph showing the training time of the individual models for 50 learning epochs is presented in Figure 5.

The test of the model was performed on 1/10 of the dataset, while training was performed on the remaining part. In the case of 10CV, 10 models were built, and hence the run time was 10 times longer than the training and test of a single model. Three repetitions of cross-validation resulted in further extension of the computation time.

The results of the 3 × 10CV for the selected classifiers (Orig_) are shown in Table 2. Table S3 in the Supplementary Materials presents these results in detail, for each individual fold.

The analysis of these results allowed us to conclude that the best classification quality was achieved for the VGG models (97.88% of accuracy). Still, DenseNet, ResNet, InceptionV3, and SqueezeNet also gave similarly good results (above 97% accuracy). Only the AlexNet-based models produced slightly inferior results (91.78% accuracy).

Based on the standard deviation values for accuracy in Table 2, one notices that the learning process for the AlexNet network stabilized to a lesser degree compared to the other network models during 100 learning epochs. It is possible that longer training could achieve better learning accuracy.

## 4. Discussion

A pilot study on pollen recognition of the same three taxa was conducted based on morphological features measured manually [34]. The values of these features were read under a microscope for 225 pollen grains (75 grains of each of the analyzed taxa). The decision tree was used as a classifier, and 94% accuracy was achieved using the best subset of features. The present study applies deep neural networks to pollen taxa directly from microscopic images of pollen grains, without the need for earlier feature extraction. Moreover, the accuracy achieved was more than 97% for five out of six of the pre-trained CNNs used in this research.

It is difficult to compare our research with most of the studies presented in the literature review, due to different classifiers and the different levels of similarity between the investigated taxa.

In this article, we evaluate the effectiveness of some CNNs by comparing their accuracy in recognizing pollen grains from microscopic images collected in the ABCPollen database. In [13,14] the authors used only one deep learning model—AlexNet—while this study considers and compares several networks, similarly to [15].

The authors of [12] assessed the performance of two deep learning networks—AlexNet and SmallerVGGNet—obtaining better results for the first model. These two classifiers have been trained from scratch, while our models are adaptations of pre-trained CNNs. Moreover, AlexNet has the simplest structure compared to the other five pre-trained networks used in our research. In light of our results, we conclude that simpler architectures lead to poorer accuracy in recognizing pollen grains from microscopic images.

In [15]—similar to this article—fine-tuned CNNs, pre-trained on the ImageNet dataset, were used. In our research, we tried to provide greater similarity of the pre-training and the target datasets. This was done by applying the additional dataset of pollen grain images—Pollen13K—for pre-training purposes. This operation did not increase the accuracy of the models presented in this paper. However, we still think that this method might be successful in other experimental setups.

In this study, most of the applied models achieved very high accuracy without any data augmentation. However, we distinguished only between the three taxa. In the case of analyzing more taxa, the performance of the models can be further improved by augmentation techniques, which allow for more training samples. This solution was applied among others in [14,15], where these operations were necessary as some classes consisted of a small number of instances.

## 5. Conclusions and Future Work

The results show that the proposed SimpleModel achieves 80% accuracy in the classification of the taxa. These results are considerable, given the difficult task of classifying objects directly from images. It is worth stressing that the differences in shape and size of the analyzed pollen grains of these taxa are minor. At the same time, some available images were not sharp, and hence the classification was further hindered.

Transfer learning methods allow for the application of pre-trained deep neural networks to other classification tasks. The best result of the 10CV was 97.88%, for the VGG model pre-trained on ImageNet data and fine-tuned on ABCPollen microscopic images. Additionally, we achieved more than 97% accuracy for each of the studied architectures, except for AlexNet.

The results indicate that the models pre-trained on ImageNet data produce a higher quality of taxon recognition based on microscopic images of pollen grains than the models pre-trained from the Pollen13K dataset. This finding is somewhat surprising, considering that the objects in the ABCPollen dataset are more similar to those from the Pollen13K dataset than to those from the ImageNet dataset.

An extension to this study could be to check whether longer training and other setups of the models using the Pollen13K dataset would obtain better input classifiers for microscopic pollen images.

Another area for future research could be to determine the impact of various image pre-processing techniques, on both the classification results and the automation of pollen detection and segmentation of single grains in the pictures. Our future plans are to collect a database of microscopic images of fresh pollen grains, check the performance of the built models using these new data, and further develop the network architecture. Continued work on identifying pollen taxa will also include automated microscope control and focus adjustment to particular grains.

## Figures and Tables

**Table 1:** Classification results for the transfer learning models.

	Model	Feature Extraction	Fine-Tuning
Type1	Orig_AlexNet	90.62%	96.09%
	Orig_ResNet	90.62%	98.44%
	Orig_VGG	NA ^1^	97.66%
	Orig_SqueezeNet	NA ^1^	99.22%
	Orig_DenseNet	NA ^1^	99.22%
	Orig_InceptionV3	NA ^1^	99.22%
Type2	ScratchPollen13K_AlexNet	51.56%	85.94%
	ScratchPollen13K_ResNet	69.53%	92.19%
Type3	FinetunedPollen13K_AlexNet	88.28%	94.53%
	FinetunedPollen13K_ResNet	89.84%	98.44%

^1^ No experiment was conducted.

**Table 2:** Results of 3 × 10CV of the models pre-trained on ImageNet images and fine-tuned on ABCPollen microscopic images.

Model	Average Accuracy	Std Dev of Accuracy
Orig_AlexNet	91.78%	0.00653
Orig_ResNet	97.61%	0.00260
Orig_VGG	97.88%	0.00173
Orig_SqueezeNet	97.21%	0.00201
Orig_DenseNet	97.71%	0.00357
Orig_InceptionV3	97.49%	0.00387

## Data Availability

Link to the ABCPollen dataset: http://kzmi.up.lublin.pl/~ekubera/ABCPollen.zip (accessed on 18 May 2021).

## Supplementary Materials

The following are available online at https://www.mdpi.com/article/10.3390/s21103526/s1; Table S1: The results of chosen models trained using the Pollen13K dataset; Table S2: The comparison of SimpleModel accuracies for the filter size; Table S3: The detailed outcomes of 3 × 10CV of the models pre-trained on ImageNet images and fine-tuned on ABCPollen microscopic images.
