# Supplementary material for: Deep Learning Methods for Improving Pollen Monitoring
Source: Sensors (Basel). 2021 May 19;21(10):3526. doi: 10.3390/s21103526 (PMC8159113; doi:10.3390/s21103526)
Supplement: Supplementary file 1 [file sensors-21-03526-s001.zip › sensors-1221259-supplementary.pdf]

Table S1. The results for chosen models trained using the Pollen13K dataset

Model	Accuracy for Pollen13K Data
FinetunedPollen13K_AlexNet	95.43%
ScratchPollen13K_AlexNet	91.81%
FinetunedPollen13K_ResNet	95.53%
ScratchPollen13K_ResNet	93.47%

Table S2. The comparison of SimpleModel accuracies for the filter size.

	Filter Size 3 × 3	Filter Size 4 × 4	Filter Size 5 × 5
**Final accuracy on test set (after 100 epochs)**	68%	**80%**	72%
**The best accuracy on test set**	76%	**80%**	78%

Table S3. The detailed outcomes of 3 × 10CV of the models pre-trained on ImageNet images and fine-tuned on ABCPollen microscopic images.

		Repetition 1		Repetition 2		Repetition 3	
		Avg Accuracy	Std Dev of Accuracy	Avg Accuracy	Std Dev of Accuracy	Avg Accuracy	Std Dev of Accuracy
Orig_AlexNet	Fold 1	83.83%	0.038	85.63%	0.036	93.83%	0.012
	Fold 2	93.36%	0.026	93.91%	0.023	93.91%	0.005
	Fold 3	94.61%	0.015	88.13%	0.011	89.84%	0.006
	Fold 4	93.67%	0.007	90.78%	0.025	92.27%	0.015
	Fold 5	89.45%	0.029	93.36%	0.008	90.70%	0.007
	Fold 6	94.38%	0.005	95.31%	0.018	88.36%	0.017
	Fold 7	87.73%	0.007	92.89%	0.006	95.39%	0.009
	Fold 8	90.16%	0.012	92.73%	0.025	90.00%	0.009
	Fold 9	87.19%	0.025	89.53%	0.008	92.97%	0.016
	Fold 10	91.33%	0.011	89.77%	0.006	93.36%	0.013
Orig_ResNet	Fold 1	98.13%	0.011	96.25%	0.010	95.47%	0.006
	Fold 2	96.09%	0.008	99.22%	0.006	97.58%	0.002
	Fold 3	98.44%	0.015	97.27%	0.004	93.20%	0.004
	Fold 4	97.03%	0.007	99.14%	0.002	98.05%	0.016
	Fold 5	99.22%	0.000	96.48%	0.006	97.27%	0.023
	Fold 6	93.28%	0.015	94.92%	0.004	100.00%	0.000
	Fold 7	96.80%	0.009	95.94%	0.005	95.70%	0.004
	Fold 8	97.03%	0.003	98.67%	0.006	96.25%	0.005
	Fold 9	97.27%	0.013	98.91%	0.005	96.88%	0.000
	Fold 10	97.73%	0.004	96.02%	0.009	97.97%	0.005
Orig_VGG	Fold 1	99.22%	0.000	96.25%	0.006	98.44%	0.000
	Fold 2	99.06%	0.003	97.11%	0.007	96.72%	0.005
	Fold 3	99.30%	0.007	98.13%	0.011	99.30%	0.006
	Fold 4	98.59%	0.008	98.44%	0.000	96.95%	0.002
	Fold 5	98.36%	0.002	98.13%	0.005	96.41%	0.004
	Fold 6	97.11%	0.005	94.38%	0.006	96.95%	0.002
	Fold 7	93.98%	0.004	97.73%	0.002	96.17%	0.004
	Fold 8	96.64%	0.004	97.73%	0.012	96.09%	0.005
	Fold 9	96.88%	0.000	96.80%	0.004	97.50%	0.005
	Fold 10	97.81%	0.003	96.25%	0.003	98.05%	0.006

Orig_SqueezeNet	Fold 1	96.95%	0.002	93.05%	0.028	96.33%	0.004
	Fold 2	95.31%	0.008	95.31%	0.000	96.88%	0.000
	Fold 3	95.47%	0.010	97.66%	0.007	96.88%	0.000
	Fold 4	99.84%	0.003	98.44%	0.000	95.31%	0.000
	Fold 5	96.09%	0.000	96.41%	0.009	99.22%	0.000
	Fold 6	98.28%	0.003	98.52%	0.002	96.17%	0.006
	Fold 7	97.11%	0.004	94.61%	0.008	96.80%	0.002
	Fold 8	96.95%	0.002	98.44%	0.000	96.41%	0.004
	Fold 9	96.88%	0.000	95.16%	0.024	98.44%	0.000
	Fold 10	95.78%	0.008	94.77%	0.005	96.95%	0.002
Orig_DenseNet	Fold 1	97.27%	0.004	96.72%	0.006	95.78%	0.010
	Fold 2	98.20%	0.004	97.19%	0.004	98.75%	0.004
	Fold 3	97.19%	0.007	99.61%	0.004	94.30%	0.021
	Fold 4	97.58%	0.002	97.89%	0.005	99.14%	0.006
	Fold 5	96.48%	0.008	96.17%	0.009	96.33%	0.056
	Fold 6	97.19%	0.008	95.23%	0.004	98.28%	0.003
	Fold 7	97.58%	0.015	98.36%	0.006	96.48%	0.004
	Fold 8	94.61%	0.032	95.31%	0.004	97.73%	0.008
	Fold 9	98.52%	0.002	99.06%	0.005	96.95%	0.014
	Fold 10	97.58%	0.004	96.88%	0.000	96.95%	0.011
Orig_InceptionV3	Fold 1	98.44%	0.004	97.19%	0.009	92.97%	0.011
	Fold 2	96.48%	0.006	98.59%	0.003	95.47%	0.009
	Fold 3	97.73%	0.002	98.98%	0.004	98.44%	0.006
	Fold 4	96.88%	0.007	98.28%	0.005	98.05%	0.004
	Fold 5	97.58%	0.006	97.19%	0.010	96.80%	0.012
	Fold 6	96.88%	0.012	97.42%	0.007	96.48%	0.010
	Fold 7	97.81%	0.013	95.31%	0.007	97.58%	0.011
	Fold 8	97.66%	0.004	96.17%	0.007	94.38%	0.005
	Fold 9	97.19%	0.022	96.64%	0.006	95.78%	0.019
	Fold 10	97.19%	0.008	94.84%	0.008	98.44%	0.005
